# Diabetes‐induced microvascular complications at the level of the spinal cord: a contributing factor in diabetic neuropathic pain

**DOI:** 10.1113/JP275067

**Published:** 2018-07-11

**Authors:** N. Ved, M. E. Da Vitoria Lobo, S. M. Bestall, C. L. Vidueira, N. Beazley‐Long, K. Ballmer‐Hofer, M. Hirashima, D. O. Bates, L. F. Donaldson, R. P. Hulse

**Affiliations:** ^1^ Cancer Biology, Division of Cancer and Stem Cells School of Medicine University of Nottingham Nottingham NG7 2UH UK; ^2^ Institute of Ophthalmology 11–43 Bath St London EC1V 9EL UK; ^3^ Department of Physiology, Anatomy and Genetics University of Oxford Oxford UK; ^4^ Arthritis Research UK Pain Centre and School of Life Sciences, The Medical School QMC University of Nottingham Nottingham NG7 2UH UK; ^5^ Paul Scherer Institute Villingen 5232 Switzerland; ^6^ Division of Vascular Biology Kobe University Japan; ^7^ Centre of Membrane and Protein and Receptors (COMPARE) University of Birmingham, Birmingham and University of Nottingham Nottingham UK; ^8^ School of Science and Technology Nottingham Trent University Nottingham NG11 8NS UK

**Keywords:** pain, diabetes, endothelial

## Abstract

**Key points:**

- Diabetes is thought to induce neuropathic pain through activation of dorsal horn sensory neurons in the spinal cord.
- Here we explore the impact of hyperglycaemia on the blood supply supporting the spinal cord and chronic pain development.
- In streptozotocin‐induced diabetic rats, neuropathic pain is accompanied by a decline in microvascular integrity in the dorsal horn. Hyperglycaemia‐induced degeneration of the endothelium in the dorsal horn was associated with a loss in vascular endothelial growth factor (VEGF)‐A_165_b expression. VEGF‐A_165_b treatment prevented diabetic neuropathic pain and degeneration of the endothelium in the spinal cord.
- Using an endothelial‐specific VEGFR2 knockout transgenic mouse model, the loss of endothelial VEGFR2 signalling led to a decline in vascular integrity in the dorsal horn and the development of hyperalgesia in VEGFR2 knockout mice.
- This highlights that vascular degeneration in the spinal cord could be a previously unidentified factor in the development of diabetic neuropathic pain.

**Abstract:**

Abnormalities of neurovascular interactions within the CNS of diabetic patients is associated with the onset of many neurological disease states. However, to date, the link between the neurovascular network within the spinal cord and regulation of nociception has not been investigated despite neuropathic pain being common in diabetes. We hypothesised that hyperglycaemia‐induced endothelial degeneration in the spinal cord, due to suppression of vascular endothelial growth factor (VEGF)‐A/VEGFR2 signalling, induces diabetic neuropathic pain. Nociceptive pain behaviour was investigated in a chemically induced model of type 1 diabetes (streptozotocin induced, insulin supplemented; either vehicle or VEGF‐A_165_b treated) and an inducible endothelial knockdown of VEGFR2 (tamoxifen induced). Diabetic animals developed mechanical allodynia and heat hyperalgesia. This was associated with a reduction in the number of blood vessels and reduction in Evans blue extravasation in the lumbar spinal cord of diabetic animals *versus* age‐matched controls. Endothelial markers occludin, CD31 and VE‐cadherin were downregulated in the spinal cord of the diabetic group *versus* controls, and there was a concurrent reduction of VEGF‐A_165_b expression. In diabetic animals, VEGF‐A_165_b treatment (biweekly i.p., 20 ng g^−1^) restored normal Evans blue extravasation and prevented vascular degeneration, diabetes‐induced central neuron activation and neuropathic pain. Inducible knockdown of VEGFR2 (tamoxifen treated *Tie2CreER^T2^*‐*vegfr2^flfl^* mice) led to a reduction in blood vessel network volume in the lumbar spinal cord and development of heat hyperalgesia. These findings indicate that hyperglycaemia leads to a reduction in the VEGF‐A/VEGFR2 signalling cascade, resulting in endothelial dysfunction in the spinal cord, which could be an undiscovered contributing factor to diabetic neuropathic pain.

## Key points

- Diabetes is thought to induce neuropathic pain through activation of dorsal horn sensory neurons in the spinal cord.
- Here we explore the impact of hyperglycaemia on the blood supply supporting the spinal cord and chronic pain development.
- In streptozotocin‐induced diabetic rats, neuropathic pain is accompanied by a decline in microvascular integrity in the dorsal horn. Hyperglycaemia‐induced degeneration of the endothelium in the dorsal horn was associated with a loss in vascular endothelial growth factor (VEGF)‐A_165_b expression. VEGF‐A_165_b treatment prevented diabetic neuropathic pain and degeneration of the endothelium in the spinal cord.
- Using an endothelial‐specific VEGFR2 knockout transgenic mouse model, the loss of endothelial VEGFR2 signalling led to a decline in vascular integrity in the dorsal horn and the development of hyperalgesia in VEGFR2 knockout mice.
- This highlights that vascular degeneration in the spinal cord could be a previously unidentified factor in the development of diabetic neuropathic pain.

## Introduction

Diabetes mellitus leads to an array of health complications that can cause significant morbidity. In people with diabetes, neuropathic pain is common (Tesfaye *et al*. 2013), characterised by enhanced responses to noxious (painful) stimuli (hyperalgesia) as well as to innocuous stimuli (allodynia). These alterations in pain perception are due to maladaptive changes in the sensory neuronal circuitry. The plasticity of the nociceptive neuronal systems, both peripheral (Reichling & Levine, 2009) and central (Latremoliere, 2009), means that they can respond to disease and/or treatment such as in diabetes (Chen & Levine, 2001; Morgado *et al*. 2010; Tan *et al*. 2012). These responses lead to neuronal sensitisation, and in diabetes, chronic pain development. The peripheral sensory nerves are well known to be affected by hyperglycaemia, including degeneration of intra‐epidermal nerve fibre innervation patterns (Hulse *et al*. 2015) and hyper‐excitability (Chen & Levine, 2003). However, pain management in people with diabetes only provides often partial pain relief (Tesfaye *et al*. 2011, 2013). There are now focused efforts to investigate how changes in nociceptive processing in the CNS, in particular the spinal cord, are altered in diabetic neuropathic pain (Biessels *et al*. 2014; Tesfaye *et al*. 2016). Studies have identified that in diabetic rodents, sensory neurons within the spinal cord elicit exaggerated responses to sensory stimulation (Morgado *et al*. 2010; Tan *et al*. 2012). Despite this evidence for the involvement of spinal cord changes in the pathogenesis of diabetic neuropathic pain, there are few investigations into those mechanisms that may underlie the development of central sensitisation in the spinal cord in diabetes (Tan *et al*. 2012; Lee‐Kubli & Calcutt, 2014).

An important component of the nervous system is the supporting blood vessel network. A compromised vascular system is integral to the development of multiple neurological diseases (e.g. stroke, Alzheimer's disease) (Tiehuis *et al*. 2008; Vandal *et al*. 2014; Winkler *et al*. 2015). Hyperglycaemia induces extensive vascular remodelling in the nervous system (Taylor *et al*. 2015; Hardigan *et al*. 2016) as well as direct glucose toxicity on sensory neurons (Chowdhury *et al*. 2014; Hulse *et al*. 2015), with both of these contributing to neurological complications including increased susceptibility of people with diabetes to cognitive decline, stroke and peripheral ischaemic neuropathies (motor, sensory and autonomic) (Said, 2007; Hardigan *et al*. 2016). Vascular endothelial growth factor‐A (VEGF‐A) is strongly implicated in diabetic vascular disease, including driving aberrant vessel growth and increased permeability in diabetic retinopathy (Cai & Boulton, 2002) and is therefore a prime target for diabetic retinopathy treatment (Gupta *et al*. 2013). The VEGF‐A gene gives rise to a variety of VEGF‐A splice variants, differing in length and terminal sequence, leading to contrasting functions (Harper & Bates, 2008). The archetypal proangiogenic isoform is VEGF‐A_165_a and is typically associated with vascular remodelling such as in diabetic retinopathy and cancer (Perrin *et al*. 2005). The VEGF‐A_165_b isoform is predominantly found in normal tissues, areas with reduced angiogenesis (Pritchard‐Jones *et al*. 2007) and pathologies where angiogenesis is impaired (e.g. systemic sclerosis and peripheral arterial disease) (Manetti *et al*. 2011; Kikuchi *et al*. 2014; Ngo *et al*. 2014). It is known to compete with VEGF‐A_165_a for VEGFR2 binding (Bates *et al*. 2002; Cébe Suarez *et al*. 2006) and can independently act through VEGFR2, resulting in cytoprotective effects (Beazley‐Long *et al*. 2013). For example, in diabetic nephropathy VEGF‐A_165_b treatment leads to a rescue in endothelial cell survival and return to normal kidney function (Oltean *et al*. 2014), and we have previously found that indicators of altered blood vascular integrity, enhanced in the peripheral nervous system and the dorsal root ganglia (DRG), can be reversed by VEGF‐A_165_b, resulting in amelioration of pain behaviours in rats (Hulse *et al*. 2015).

Enhanced sensitivity in central nociceptive networks [spinal cord (Tan *et al*. 2012; Lee‐Kubli & Calcutt, 2014), brain (Silva *et al*. 2013)] have long been attributed to neuroplastic changes. Despite extensive evidence that the cerebral vasculature is altered in diabetic rodents, there has been limited investigation into the neurovascular interactions in the spinal cord, particularly with reference to nociception (Costigan *et al*. 2009; Beggs *et al*. 2010). Here we hypothesised that a decline (endothelial cell loss) in the vascular system within the spinal cord could contribute to the onset of diabetic neuropathic pain. Using an *in vivo* rat model of type 1 diabetes, and an inducible VEGF receptor‐2 (VEGFR2) knockdown transgenic mouse, neurovascular disruption in the spinal cord was associated with changes in neuropathic pain. Administration of the VEGF‐A_165_b isoform protected the endothelial component of the CNS, and prevented diabetic neuropathic pain.

## Methods

### Ethical approval, animals used and induction of diabetes

Twenty‐four male (21 Evans blue and 3 S1 tissue collection) and 42 female (24 Evans blue and 18 S1 tissue collection) Sprague Dawley rats (∼250 g) were used in this study. Experiments were carried out in accordance with the institution's animal welfare committee (University of Nottingham), and conform to the principles and regulations as described by Grundy (2015). Procedures were carried out in accordance with the UK Home Office Animals (Scientific Procedures) Act 1986 and EU Directive 2010/63/EU after review by the local Animal Welfare and Ethics Review Board (University of Nottingham). Diabetes was induced by i.p. injection of streptozotocin (STZ; 50 mg kg^−1^) (Hulse *et al*. 2015). Animals had *ad libitum* access to standard chow and were housed in groups (*n *> 2) under 12:12 h light–dark conditions. In male rats, experimental groups (1 week, no insulin supplementation) were naïve (sham injected *n* = 9) and STZ treated (*n* = 12). At the end of the study animal weight and blood glucose (>15 mmol) were: naïve = 339 ± 8.26 g and 6.8 ± 0.85 mmol; diabetics = 314 ± 11.86 g and 30.12 ± 0.95 mmol, respectively.

In female rats (8 week experiments), animals were treated with insulin using one‐third of an insulin pellet (LinShin, Toronto, Canada) implanted under isoflurane anaesthesia (2–3% in O_2_) (Calcutt, 2004). Experimental treatments were biweekly recombinant human (rh)VEGF‐A_165_b (20 ng g^−1^ body weight, i.p. twice weekly from week 1) or saline (vehicle; i.p.). This VEGF‐A_165_b regime has previously been used (Beazley‐Long *et al*. 2013; Hulse *et al*. 2014). Blood glucose and weight was measured in all animals at the end of the study – blood glucose: naïve = 8.08 ± 0.72 mmol, diabetic + vehicle = 29.27 ± 1.35 mmol, diabetic + VEGF‐A_165_b = 30.81 ± 0.78 mmol; animal body weight: naïve = 324.1 ± 8.5 g, diabetic + vehicle = 284.2 ± 7.3 g, diabetic + VEGF‐A_165_b = 289.1 ± 5.4 g.

Seventy‐five transgenic mice were used in this study(C57.bl6, 25–30 g; both sexes). Tie2CreER^T2^ mice [Tg(Tek‐cre/ER^T2^)1Arnd, European Mutant Mouse Archive] were crossed with *vegfr2* ^fl/fl^ (generated/used as previously described; Albuquerque *et al*. 2009; Sison *et al*. 2010; Beazley‐Long *et al*. 2018). All mice used were *vegfr2* ^fl/fl^ and either Tie2CreER^T2^ positive (*n* = 27) or Tie2CreER^T2^ negative (*n* = 28) and dosed once daily (i.p.) with 1 mg tamoxifen or vehicle (10% ethanol in sunflower oil) for five consecutive days.

### Nociceptive behaviour

Nociceptive behavioural experiments were carried out as previously described (Drake *et al*. 2014; Hulse *et al*. 2016) on 8‐week‐old diabetic animals and age matched sham controls (naïve). Mechanical withdrawal thresholds were measured using von Frey (vF) monofilaments (Hulse *et al*. 2015) or a mechanical pincher (Drake *et al*. 2014; Hirschberg *et al*. 2017). A range of vF hairs were applied to the hind paw plantar surface (a maximum of 5 s or until paw withdrawal). A total of five vF applications were applied per weighted hair and force response curves were generated and withdrawal values were calculated as the weight at which withdrawal frequency = 50%. A mechanical pincher [equipped with strain gauges and calibrated to force (g)] (Drake *et al*. 2014) was applied to the hind paw until the animal withdrew to determine mechanical hyperalgesia. Raw data were acquired through a Neurolog power unit and a bridge amp module (Digitimer, Welwyn Garden City, UK), with digital acquisition via CED micro1401v3 and Spike2 v7 software (Cambridge Electronic Design, Cambridge, UK). Withdrawal to heat was determined using the Hargreaves test (Hargreaves *et al*. 1988). The experimenter was blinded to treatment.

### Evans blue extravasation

*In vivo* vascular perfusion was evaluated using Evans blue dye, as previously described (Xu *et al*. 2001). Animals [age matched sham controls (naïve), week 1 and week 8 diabetic rats] were terminally anaesthetised (ketamine medetomidine i.v. 50 mg kg^−1^) and infused (via the external jugular vein) with Evans blue dye i.v. (Sigma‐Aldrich, Poole, UK; 45 mg kg^−1^) at 120 mmHg pressure. Two minutes after infusion, 0.2 mL arterial blood was withdrawn, followed by subsequent 0.1 mL withdrawals every 15 min for 2 h. After 2 h, 0.2 mL blood was withdrawn followed by cardiac perfusion of 50 mL saline at 120 mmHg. Lumbar spinal cord and a single brain hemisphere were excised (whole spinal cord including ventral and dorsal horn) and weighed (wet weight). Tissue was dried at 70°C overnight and weighed (dry weight). Dried tissue was incubated in 0.15 mL formamide (Sigma Aldrich) at 70°C overnight. Blood samples were centrifuged (12,000 rpm, 45 min, 4°C), and the supernatant from tissue and blood samples were analysed at 620 nm. Evans blue extravasation was calculated as: solute flux (μg min^–1^ g^–1^) = Evans blue mass (μg)/tissue dry weight (g) divided by time (120 min).

## Immunofluorescence analyses

Animals from all experimental groups (normal, diabetes, diabetes + VEGF‐A_165_b; 8 weeks) were terminally anaesthetised (sodium pentobarbital 60 mg kg^−1^ i.p.) and transcardially perfused, with PBS followed by 4% paraformaldehyde in PBS (PFA; pH 7.4). Tissue was prepared as previously described (Hulse *et al*. 2016). Spinal cords (40 μm thickness) were incubated in primary antibodies (see below) in blocking solution (5% bovine serum albumin, 10% fetal calf serum), overnight at 4°C (IB_4_ 72 h). Primary antibodies/markers and dilutions used were: biotin conjugated isolectin B_4_ (IB_4_; 1:100, Sigma‐Aldrich); rat anti‐CD31 (MEC13.3; 1:10, Santa Cruz Biotechnology, Inc., Santa Cruz, CA, USA), mouse anti‐CD31 (1:100, Abcam, Cambridge, MA, USA), rabbit anti‐GFAP (1:500, Abcam), rabbit anti‐fos (1:100, Santa Cruz); mouse anti‐NeuN (1:200; Millipore, Billerica, MA, USA), rabbit anti‐VEGFR2 (1:200, 55B11, Cell Signaling, Danvers, MA, USA), rabbit anti‐cleaved caspase 3 (1:500, Cell Signaling) and anti‐rabbit biotinylated IgG (1:500, Jackson Laboratories, West Grove, PA, USA). Secondary antibodies were incubated in PBS + 0.2% Triton X‐100, which were Alexa Fluor 488‐conjugated chicken anti‐mouse, Alexa Fluor 555‐conjugated donkey anti‐rabbit and streptavidin‐conjugated Alexa Fluor‐555 (1:500, all Invitrogen, UK). Confocal imaging of the dorsal horn of the lumbar spinal cord of all groups was performed on a Leica TCS SPE confocal microscope.

### Spinal cord endothelial cell culture

Whole lumbar spinal cords were dissected from adult male Sprague Dawley rats and dissociated (0.125% collagenase). Endothelial cells were extracted and cultured in endothelial media (M199 media, 60 μg mL^–1^ endothelial cell growth supplement and 50 μg mL^–1^ heparin). Endothelial cells were plated (1% gelatin coated) onto either six‐well plates (for protein extraction) or 96‐well plates (for cell viability studies). For cell viability assays, when 80% confluent endothelial cells were incubated for 24 h in either 5 mm glucose (normal), 50 mm mannitol (osmotic control) or 50 mm glucose (high glucose) ± 2.5 nm VEGF‐A_165_b or vehicle. Cell death was determined with neutral red (Sigma‐Aldrich).

## Flow cytometry

Spleens were isolated from control (CTL) and endothelial cell knockout (ecKO) transgenic mouse experimental groups and placed in media (containing RPMI1640, penicillin/streptomycin, 10% FBS, 1% l‐glutamine, 0.1% sodium pyruvate). Tissue was mechanically dissociated through a 40 μm cell strainer and washed through with media. Cell suspension was then centrifuged at 1500 rpm for 3 min. Supernatant was removed and cells were resuspended in 2 mL red cell lysis medium (Sigma), then left for 30 s. Following this 10 mL of medium was added and the cells were centrifuged as previously described. Cells were then fixed in 4% paraformaldehyde for 15 min at room temperature and then subsequently washed three times. Cells were permeabilised with 0.4% Triton X‐100 in PBS for 15 min at room temperature. Cells were blocked in 1% FBS in PBS, mixed gently and incubated for 30 min at room temperature. Antibodies [CD11b‐APC (1:100; BioLegend, San Diego, CA, USA) and F4/80‐PE (1:100; Biolegend)] were added, left overnight in the fridge, and samples were analysed on a MoFlo analyser.

## qPCR method

Total RNA was extracted from whole lumbar spinal cord tissue isolated from CTL and ecKO transgenic colony experimental groups using TRIzol reagent (Invitrogen). cDNA synthesis was carried using a PrimeScript RT reagent kit (TaKaRa, Shiga, Japan; RR037A) with a starting amount of 1 μg of RNA. The resulting cDNA was used for quantitative PCR using a LightCycler 480 SYBR Green I Mastermix (Roche, Indianapolis, IN, USA; 04707516001) following the manufacturer's instructions. CD31 and VE‐cadherin primers were synthesised by Eurofins. Beta actin and VEGFR2 primers were synthesised by Sigma‐Aldrich. Beta actin was used as a reference gene. The primer sequences were as follows (5′–3′):

β‐actin F – 5′ ATTGCCAATGAGCGGTTC‐3′
β‐actin R – 5′ GGATGCCACAGGACTCCA‐3′
CD31 F – 5′ GAAATGCTCTCGAAGCCCAG‐3′
CD31 R – 5′ ACCTCGAGAGTCTGGAAGTC‐3′
VE‐cadherin F – 5′ TCCCTGGACTATGAAGTCAT‐3′
VE‐cadherin R – 5′ GAAGACAGGGGGCTCATCCA‐3′
VEGFR2 F – 5′ GGATCTGAAAAGACGCTTGG‐3′
VEGFR2 R – 5′ TGCTCCAAGGTCAGGAAGTC‐3′

### Western blotting

Protein was extracted from spinal cord endothelial cells and human umbilical vein endothelial cells as well as spinal cord tissue as previously described (Vencappa *et al*. 2015; Hulse *et al*. 2016). Animals from all experimental groups (CTL and ecKO transgenic mice; normal, diabetes, diabetes + VEGF‐A_165_b; 8 weeks) were terminally anaesthetised with sodium pentobarbital (i.p. 60 mg kg^−1^, Sigma‐Aldrich). Lumbar spinal cords were extracted, frozen immediately and stored at −80°C until sample processing. Then, 100 μg endothelial cell lysate and 80 μg of each spinal cord lysate were loaded in a 4–20% precast Mini‐Protean TGX gel (BioRad, Hercules, CA, USA), separated by SDS‐PAGE and transferred using a Trans‐blot turbo transfer system (BioRad). The membrane was incubated in 5% milk powder in Tris‐buffered saline (TBS)‐Tween 0.1% (TBST) for 30 min at room temperature. Primary antibodies, mouse anti‐CD31 (2 μg ml^−1^, Abcam; AB24590), rabbit anti‐occludin (5 μg ml^−1^, Invitrogen; 71‐1500), mouse anti‐VE cadherin (5 μg ml^−1^, BD Biosciences, Franklin Lakes, NJ, USA; 550548), rabbit anti‐VEGFR2 (1:200, 55B11, Cell Signaling), rabbit anti‐Pan VEGF‐A (A20, 1 μg ml^−1^, Santa Cruz; sc‐152), mouse anti‐VEGF‐A_165_b (2 μg ml^−1^, Abcam; ab‐14994), and rabbit anti‐Actin (1:100, Santa Cruz) antibodies were diluted in blocking solution and incubated overnight at 4°C. Secondary antibodies (Licor donkey anti‐rabbit and anti‐mouse antibodies 1:10,000) in TBST‐0.1% 1% BSA and visualised on the Licor Odessey.

### Statistical analysis

All data are represented as mean ± SEM unless stated otherwise and the experimenter was blinded where appropriate. Data were acquired/quantified using Microsoft Excel 2010 and Graphpad Prism 6, and Imaris (Bitplane) Spike2 v7 software (CED) was used to digitally acquire mechanical withdrawal thresholds from the pincher and for offline analysis. Immunofluorescence was quantified by obtaining 10 random non‐sequential sections (Z stacks) per animal and a mean value was calculated per animal. Dorsal horn spinal cords (Lamina I–V) were imaged with the confocal microscope as described above, and vessels were identified through CD31 and IB_4_ immunoreactivity. IB_4_ signal fluorescence was vascular in the majority of the spinal cord with C‐fibre projections also staining in the peripheral laminae (I and II), but vessels could also be clearly delineated here by CD31 staining as well as by morphology. Stained images were rendered on Imaris 8.11 imaging software. This allows for automated quantification of vessel diameter and blood vessel volume. Neuron number (cleaved caspase‐3 and C‐Fos quantification) was determined as per laminae of the dorsal horn and therefore expressed according to lamina I–V as previously characterised (Hsieh *et al*. 2015). Western blot densitometry was quantified using the ImageJ (https://imagej.nih.gov/ij/) gel quantification plugin. Paw mechanical withdrawal thresholds, the number of blood vessels in the dorsal horn of the spinal cord and spinal cord Evans blue extravasation were analysed using a Mann–Whitney test. Evans blue, IB_4_ positive blood vessel volume and length, mechanical and heat nociceptive behavior, and western blot densitometry quantification were analysed using a Kruskal–Wallis and appropriate *post hoc* tests.

### Results

**Figure 1:**
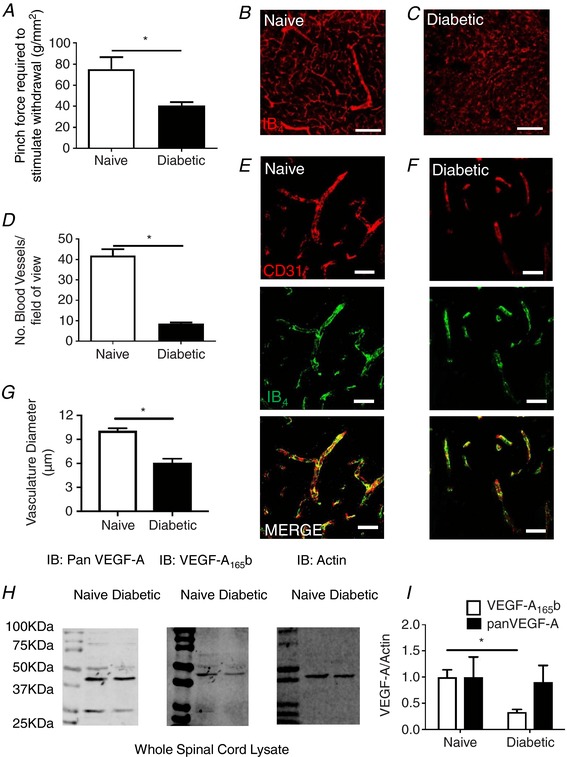
Diabetes‐induced neuropathic pain is associated with a reduction in spinal cord vasculature and a decrease in VEGF‐A_165_b expression *A*, diabetes resulted in a reduction in mechanical withdrawal threshold measured by pincher, when compared with naïve age‐matched animals (^*^ *P* < 0.05, *n* = 5 per group). *B*, blood vessels identified (IB_4_) in the deeper laminar layers of the spinal cord (layers III–VI) of the naïve animal (*C*) with a decline in vascular staining in the diabetic animal. *D*, there was a reduction in blood vessel (CD31/IB_4_+ve) number (^*^ *P* <0.05, *n* = 4 per group) [*E* = naïve, *F* = diabetic; reduced diameter (^*^ *P* < 0.05, *n* = 4 per group)] as well as diameter (*G*) in the lumbar spinal cord in diabetic animals compared with naïve controls. *H*, immunoblot of pan‐VEGF, VEGF‐A_165_b and actin in lysates from spinal cord of normal and diabetic animals. *I*, densitometry analysis demonstrates no change in pan‐VEGF‐A expression and a decrease in VEGF‐A_165_b expression in diabetic lumbar spinal cord *versus* naïve animals (^*^ *P* < 0.05, *n* = 5 per group). Scale bars: *B* and *C* = 40 μm, *E* and *F*= 20 μm. [Color figure can be viewed at http://wileyonlinelibrary.com]

Diabetes resulted in increased mechanical hyperalgesia – a reduction in withdrawal threshold to a noxious stimulus (hindpaw pinch) when compared to both before diabetes (*P *< 0.05) and to vehicle/age matched animals at week 8 after STZ injection (naïve; Fig. 1 *A*, ^*^ *P *< 0.05). This was accompanied by a reduction in the number of blood vessels in the dorsal horn of the lumbar region of the spinal cord (Fig. 1 *B–D*, ^*^ *P *< 0.05) as well as a reduction in microvessel diameter (labelled with CD31 and IB_4_) (Fig. 1 *E–G*). The VEGF‐A family is a key regulator of angiogenic processes. Pan‐VEGF‐A expression was unaltered in the lumbar spinal cord of diabetic rats when compared to age/gender matched control animals (Fig. 1 *H* and *I*). However, VEGF‐A_xxx_b expression was significantly reduced in the lumbar spinal cord of diabetic animals (Fig. 1 *H* and *I*, ^*^ *P *< 0.05).

**Figure 2:**
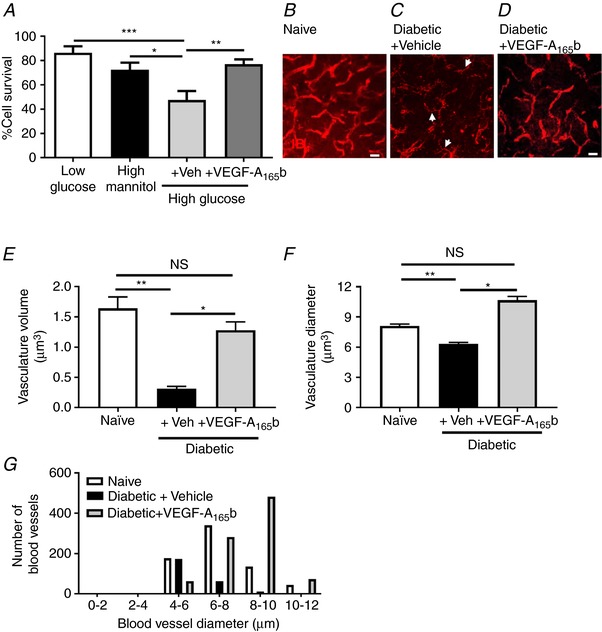
Diabetes‐induced vascular impairment in the spinal cord *A*, isolated spinal cord endothelial cells demonstrated increased cell death in 50 mm glucose when compared with 50 mm mannitol and 5 mm glucose (^*^ *P* < 0.05, ^***^ *P* < 0.001). VEGF‐A_165_b treatment prevented high glucose‐induced endothelial cell death (^**^ *P* < 0.01). *B*, IB_4_ stained vasculature in the spinal cord of naïve age matched controls was compared with that in (*C*) diabetic + vehicle (arrowheads = vessels smaller than 6 μm) and (*D*) diabetic + VEGFA_165_b. *E*, there was a significant reduction in total volume of the microvasculature in the spinal cord of the diabetic + vehicle group in addition to (*F*) a reduction in vessel diameter compared with naïve controls (^**^ *P* < 0.01, ^***^ *P *< 0.001, *n* = 4 per group). *E* and *F*, VEGF‐A_165_b treatment prevented the diabetes‐induced vascular degeneration in the lumbar spinal cord (^**^ *P* < 0.01, *n* = 4 per group). *G*, VEGF‐A_165_b treatment also prevents the diabetes‐induced decrease in larger and intermediate microvessels. Scale bar: *B–D* = 25 μm. [Color figure can be viewed at http://wileyonlinelibrary.com]

**Figure 3:**
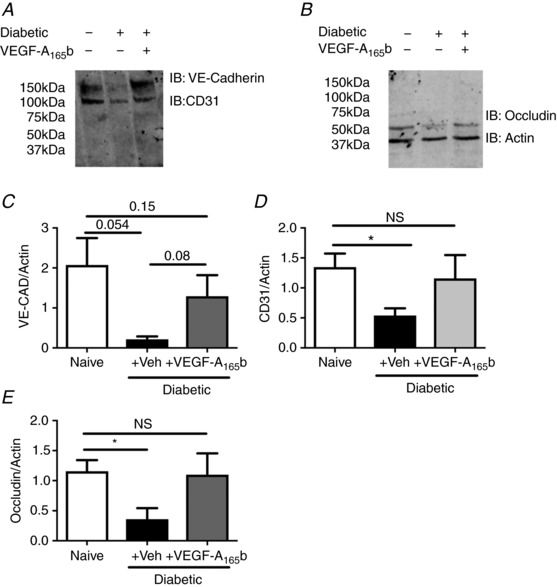
Diabetes‐induced degeneration of the endothelium Immunoblots using dual colour far red imaging for endothelial markers (*A*, VE‐cadherin, CD31; and *B*, occludin and actin) demonstrated (*C*) a non‐significant reduction in VE‐cadherin expression and significant reductions in (*D*) CD31 and (*E*) occludin in the diabetic + vehicle group compared with naïve and diabetic + VEGF‐A_165_b animals (^*^ *P* < 0.05, *n* = 5).

As hyperglycaemia affected spinal cord microvasculature and reduced VEGF‐A_165_b expression, we investigated the direct cytoprotective actions of VEGF‐A_165_b upon cultured spinal cord endothelial cells. Isolated spinal cord endothelial cells showed increased cell death when cultured in high (50 mm) *versus* low glucose (5 mm) conditions (Fig. 2 *A*). There was no effect of 50 mm mannitol, an osmotic control. VEGF‐A_165_b treatment prevented high glucose‐induced endothelial cell death (Fig. 2 *A*). Consequently, the cytoprotective actions of VEGF‐A_165_b on the spinal cord endothelium were investigated *in vivo*. The vascular network was well defined in naïve animals (Fig. 2 *B*) and was reduced in number in diabetic + vehicle treated animals (Fig. 2 *C*), but this was prevented by VEGF‐A_165_b (Fig. 2 *D*). Quantification indicated a reduction in endothelium (reduced total vascular volume) within the dorsal horn of the lumbar region of the spinal cord, which was rescued by VEGF‐A_165_b (Fig. 2 *E*). There was also an overall reduction in dorsal horn vessel diameter, again prevented by VEGF‐A_165_b (Fig. 2 *F*). There was a non‐significant increase in vessel diameter in the diabetic + VEGF‐A_165_b treated group when compared with the naïve group (Fig. 2 *F*). Analysis of the frequency distribution of the vessels by size indicated that the reduction in size in the diabetic animals was due to a reduced number of the ‘larger microvessels’ (8–12 μm) rather than a reduction in the number of small vessels (<8 μm) (Fig. 2 *G*). To determine whether markers of endothelial integrity/activation (including junctional markers) were altered in the lumbar spinal cord in diabetes, lumbar spinal cord protein samples were subjected to immunoblotting for VE‐cadherin (Fig. 3 *A* upper band), CD31 (Fig. 3 *A* lower band), and occludin (Fig. 3 *B* upper band; lower band actin). There was a marked reduction in junctional and adhesion molecules in diabetic + vehicle treated rats, with non‐significant reductions in VE‐cadherin (Fig. 3 *C*) and significant reductions in CD31 (Fig. 3 *D*) and occludin (Fig. 3 *E*), all of which were prevented by VEGF‐A_165_b treatment.

**Figure 4:**
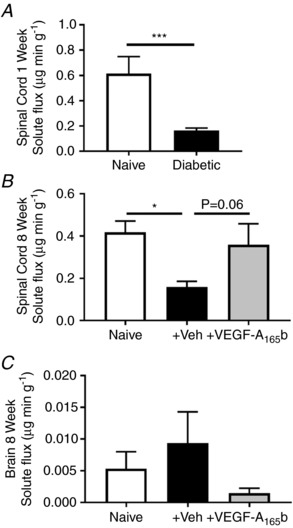
Reduced vascular functionality in the spinal cord of diabetic rats There was a significant reduction in Evans blue solute flux in the lumbar spinal cord of diabetic animals after (*A*) 1 week (naïve, *n* = 9; diabetes, *n* = 12; ^***^ *P* < 0.001) and (*B*) 8 weeks (^*^ *P* < 0.05, *n* = 4/5 per group). VEGF‐A_165_b treatment prevented the diabetes‐induced reduction in solute flux within the lumbar spinal cord at 8 weeks (^*^ *P* < 0.05, *n* = 4/5 per group). *C*, there was no change in solute flux in the brain of any treatment group (naïve *vs*. diabetic + vehicle: *P* = 0.52; diabetic + vehicle *vs*. diabetic + VEGF‐A_165_b: *P* ≥ 0.99; *n* = 4/5 per group).

To investigate the possible functional changes in the microvasculature within the spinal cord in diabetes, Evans blue extravasation was measured (Xu *et al*. 2001). Evans blue vascular leakage is dependent on blood flow, surface area, hydrostatic pressure and vascular permeability. In an acute diabetic rodent model (1 week) there was a pronounced decrease in Evans blue extravasation within the lumbar region of the spinal cord compared with that of the control cohort (Fig. 4 *A*). Diabetic groups were systemically (i.p.) treated with either saline or VEGF‐A_165_b (20 ng g^−1^) using a longer term diabetic model (8 weeks). In longer duration diabetes (8 weeks) there was also a significant reduction in Evans blue extravasation compared with the control naive group (Fig. 4 *B*), which was prevented by VEGF‐A_165_b (Fig. 4 *B*). Brains were also extracted from the long term (8 week) study (Fig. 4 *C*) although there was no difference in solute flux between groups.

**Figure 5:**
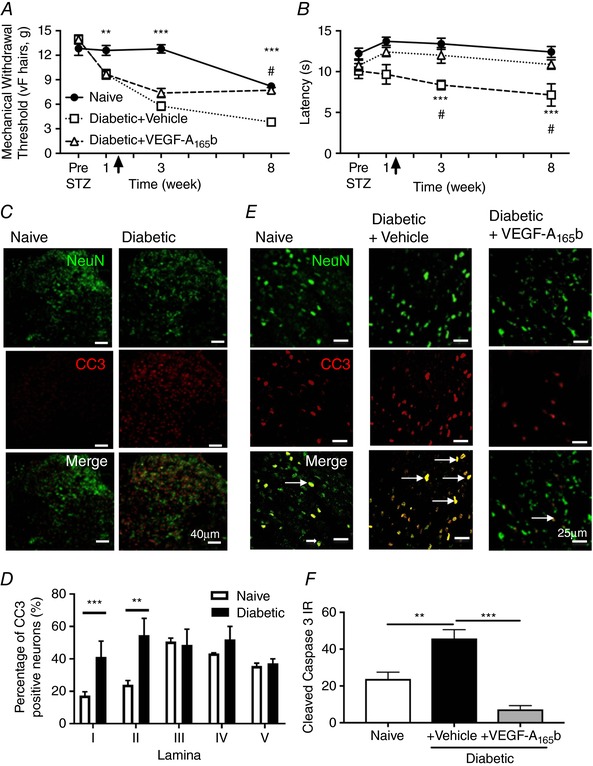
Diabetes‐induced dysfunction of microvasculature in the spinal cord and neuropathic pain is reversed by VEGF‐A_165_b *A*, diabetic + vehicle animals demonstrated a decrease in mechanical withdrawal threshold (vF hairs) and (*B*) reduced withdrawal latency to heat compared with both naïve and diabetic + VEGF‐A_165_b treated groups (^**^ *P* < 0.01, ^***^ *P* < 0.001 naïve *vs*. diabetic + vehicle; ^#^ *P *< 0.001 diabetic + VEGF‐A_165_b *vs*. diabetic + vehicle, *n* = 5 per group). Arrow highlights onset of VEGF‐A_165_b treatment. *C*, cleaved caspase‐3 (red = CC3) and sensory neuron (green = NeuN) staining in the spinal cord (scale bar = 40 μm). *D*, there was an increase in CC3 expression in sensory neurons in the superficial lamina (I and II) of the dorsal horn of the spinal cord in the diabetic + vehicle groups compared with naïve age‐matched controls and VEGF‐A_165_b treated diabetic animals. *E*, there was an increased number of CC3‐positive dorsal horn sensory neurons (arrows) in the dorsal horn of the spinal cord in the diabetic + vehicle groups compared with naïve age‐matched controls and VEGF‐A_165_b treated diabetic animals. This is graphically represented in (*F*) (^*^ *P* < 0.05, *n* = 4). [Color figure can be viewed at http://wileyonlinelibrary.com]

**Figure 6:**
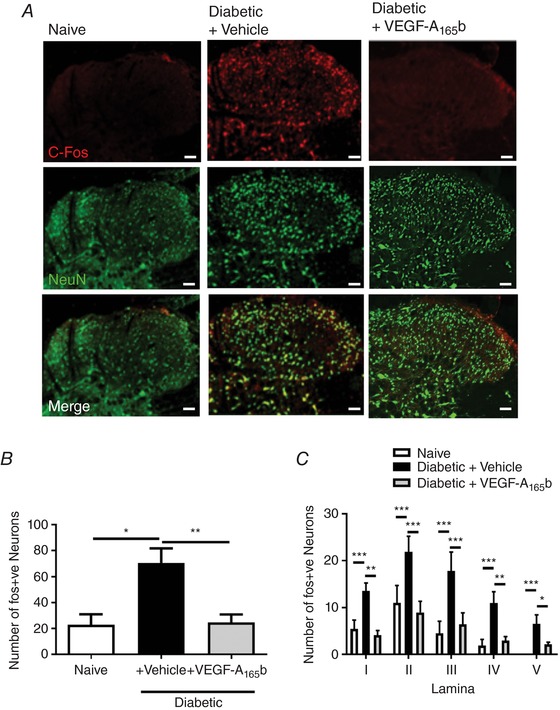
Diabetes‐induced hyperactivity in sensory neurons of the dorsal horn in the spinal cord *A* and *B*, immunoreactivity of a marker of central sensitisation, c‐fos (red; NeuN green), was increased in sensory neurons in the dorsal horn of the spinal cord in diabetic + vehicle animals *versus* naïve animals. This was reduced by VEGF‐A_165_b treatment. *C*, there was an increase of c‐fos expression in sensory neurons in all lamina of the dorsal horn (I–V) in the diabetic + vehicle group when compared to naïve and diabetic + VEGF‐A_165_b groups (^*^ *P* < 0.05, ^**^ *P* < 0.001, ^***^ *P* < 0.001; *n* = 4). [Color figure can be viewed at http://wileyonlinelibrary.com]

Mechanical hypersensitivity and heat hyperalgesia developed only in the diabetic + vehicle animals. VEGF‐A_165_b treatment (timepoint of administration shown via arrow in Fig. 5 *A* and *B*) not only prevented the spinal cord vascular degeneration (consistent with previous experiments; Hulse *et al*. 2015; Ved *et al*. 2017) but also prevented diabetic neuropathic pain behaviours (mechanical allodynia, Fig. 5 *A*; and heat hyperalgesia, Fig. 5 *B*). Within the spinal cord, not only was the vasculature disturbed in the diabetic + vehicle group, but sensory neurons (NeuN) in the dorsal horn also expressed increased cleaved caspase‐3 (CC3), an indicator of neuronal damage, when compared with naïve age matched animals (Fig. 5 *C*). There was increased CC3 immunoreactivity in the superficial lamina (I and II) of the dorsal horn of diabetic animals (Fig. 5 *D*). This was blocked by VEGF‐A_165_b treatment (Fig. 5 *E* and *F*). Sensory neurons within the spinal cord, once activated, express the immediate early gene c‐fos (Hunt *et al*. 1987). c‐fos is a marker of neuronal activation in chronic pain states (Kalynovska *et al*. 2017; Khasabov *et al*. 2017) and is an indicator of spinal neuronal activation in diabetic neuropathic pain (Morgado *et al*. 2010). There was an increase in c‐fos expression in neurons (NeuN co‐labelled) within the lumbar dorsal horn in the spinal cord in diabetic rats (Fig. 6 *A* and *B*) compared with naïve animals. Systemic treatment with VEGF‐A_165_b led to an attenuation of the diabetes induced c‐fos expression (Fig. 6 *A* and *B*). All lamina of the dorsal horn demonstrated increased c‐fos expression in neurons in the diabetic + vehicle group compared with the naïve and diabetic + VEGF‐A_165_b groups (Fig. 6 *C*).

**Figure 7:**
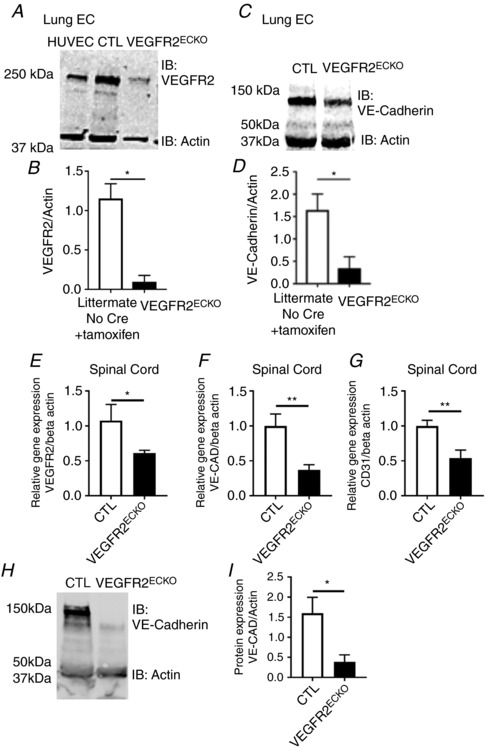
Tamoxifen induced VEGFR2 knockout VEGFR2 was inducibly knocked out in endothelial cells by crossing *vegfr2* ^fl/fl^ with Tie2Cre^ERT2^ mice and treating with tamoxifen (VEGFR2^ECKO^). *A*, in lung tissue, VEGFR2 protein was detected in tamoxifen‐treated *vegfr2* ^fl/fl^ mice lacking Cre (CTL), but not in VEGFR2^ECKO^ mice. *B*, immunoblot densitometry demonstrated reduced VEGFR2 protein in the VEGFR2^ECKO^ mice compared with controls (^*^ *P*<0.05, *n* = 6 per group). *C* and *D*, VE‐cadherin expression was also reduced in lung tissue of the VEGFR2^ECKO^ mice (^*^ *P* < 0.05, *n* = 6 per group). *E*, there was a reduction in VEGFR2 in the spinal cord of the VEGFR2^ECKO^ mice when compared to CTL mice (^*^ *P* < 0.05, *n* = 4 per group. This was accompanied by reductions in endothelial markers (*F*) VE‐cadherin and (*G*) CD31 (^**^ *P* < 0.01, *n* = 4 per group). *H*, western blot of the lumbar spinal cord from VEGFR2^ECKO^ mice demonstrating (*I*) a reduction in VE‐cadherin expression when compared to CTL mice.

The involvement of VEGF‐A/VEGFR2 signalling on spinal cord endothelial cell function and survival and the relationship to the generation of behavioural hypersensitivity was determined by using an endothelial cell‐specific inducible VEGFR2 knockdown *in vivo*. Systemic tamoxifen treatment led to a reduction in VEGFR2 protein expression in endothelial cells from *vegfr2* ^flfl^ Tie2CreER^T2^‐positive mice, i.e. VEGFR2 endothelial cell knockout (VEGFR2^ECKO^) compared with control mouse endothelial cells (CTL = *vegfr2* ^flfl^ Tie2CreER^T2^‐negative + tamoxifen) isolated from lung (Fig. 7 *A*–*B*), as well as a reduction in VE‐cadherin expression (Fig. 7 *C* and *D*). In the spinal cord there was a reduction in VEGFR2 (Fig. 7 *E*) as well as VE‐cadherin (Fig. 7 *F*) and CD31 (Fig. 7 *G*) expression in the VEGFR2^ECKO^ animals compared with CTL animals. In addition, VE‐cadherin protein expression from the spinal cord of VEGFR2^ECKO^ animals was reduced when compared with the CTL animals (Fig. 7 *H* and *I*).

**Figure 8:**
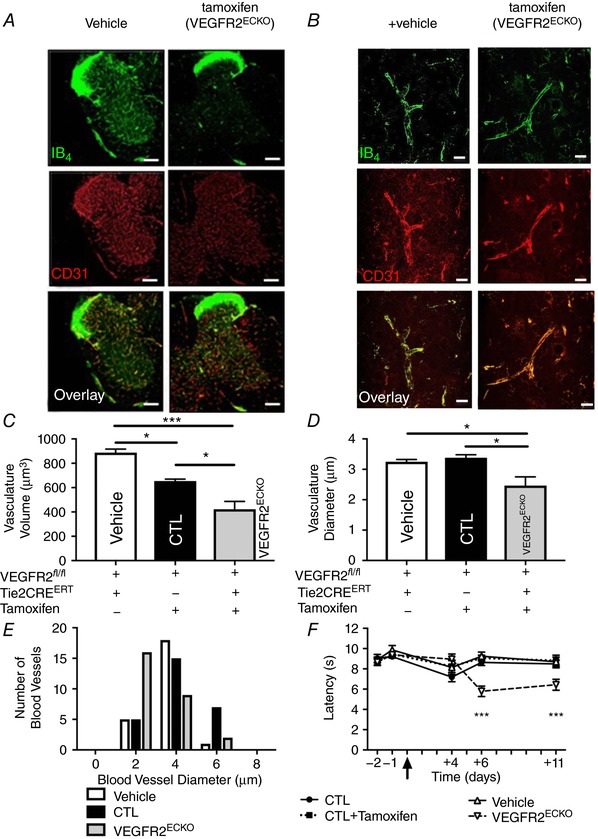
Inducible endothelial cell *vegfr2* knockout caused microvasculature loss in the dorsal horn of the lumbar region of the spinal and hyperalgesia Representative images from the microvessels in (*A*, lower power; *B*, high power) *vegfr2^f^* ^l/fl^ Tie2Cre^ERT2^ positive mice + vehicle (Vehicle) and (*D*–*F*) VEGFR2^ECKO^. VEGFR2^ECKO^ mice had a significant reduction in microvasculature (*C*) volume and (*D*) diameter compared with controls (CTL and vehicle). (^***^ *P* < 0.000, ^*^ *P* < 0.01; comparison between Vehicle and CTL vessel diameter *P* = 0.5391). *E*, VEGFR2^ECKO^ mice had an increased number of smaller microvessels *versus* other experimental groups. *F*, VEGFR2^ECKO^ mice showed a reduced withdrawal latency to heat when compared to control mice [^***^ *P* < 0.001; comparison made at day 11 between CTL (*P* = 0.003), Vehicle (*P* = 0.001) and CTL + Vehicle (*P* = 0.0008) against VEGFR2^ECKO^ mice, *n* = 10/11 per group]. [Color figure can be viewed at http://wileyonlinelibrary.com]

When compared with CTL mice (Fig. 8 *A*–*C*), the VEGFR2^ECKO^ animals (Fig. 8 *A*, low power; Fig. 8 *B*, high power) showed a significant decline in vascular integrity within the lumbar spinal cord 8 days after the final drug injection. The endothelium demonstrated a reduced endothelial volume (Fig. 8 *C*) as well as a reduced diameter of spinal cord microvessels in the VEGFR2^ECKO^ mice treated with tamoxifen when compared with CTL mice (and VEGFR2^fl/fl^ with Cre but not given tamoxifen ‘vehicle’) (Fig. 8 *D*). There was an increase the number of smaller vessels in the VEGFR2^ECKO^ mice when compared to control mice (CTL and vehicle) (Fig. 8 *E*).

**Figure 9:**
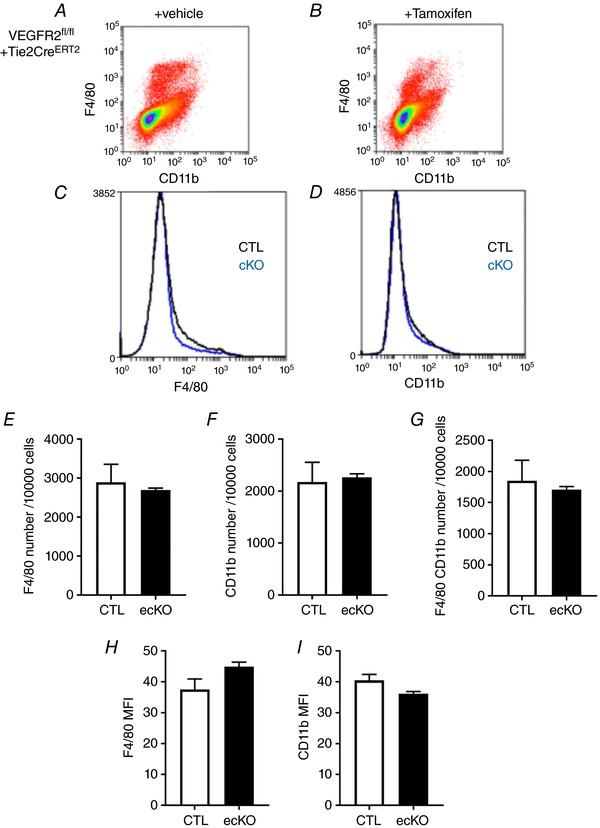
Tamoxifen did not induce a loss in haematopoietic cells in the mouse spleen *A* and *B*, cells isolated from mouse spleens were analysed using flow cytometry to identify (*C*) F4/80 and (*D*) CD11b cell populations. In VEGFR2^ECKO^ + tamoxifen mice there were no changes in cell number in (*E*) F4/80 (*P* = 0.630), (*F*) CD11b (*P *> 0.99) and (*G*) F4/80/CD11b (*P *> 0.99) cell populations or (*H* and *I*) median fluorescence intensity (F4/80, *P* = 0.11; CD11b, *P* = 0.11) when compared with tamoxifen‐treated Tie2CRE mice (*n* = 4 per group). [Color figure can be viewed at http://wileyonlinelibrary.com]

In VEGFR2^ECKO^ and CTL mice there was no difference in nociceptive behaviour prior to tamoxifen administration (Fig. 8 *F*). The *vegfr2* ^flfl^ Tie2CreER^T2^ mice treated with vehicle (‘vehicle’) and CTL mice treated with either vehicle or tamoxifen demonstrated no change in nociceptive behavioural responses to heat (Fig. 8 *F*). However, the VEGFR2^ECKO^ mice demonstrated a pronounced heat hypersensitivity compared with all other groups following tamoxifen injection (Fig. 6 *I*). To exclude the involvement of a subset of haemopoietic cells that express Tie2 and VEGFR2 we determined the impact of VEGFR2 knockdown in this cell population. There was no change in the number of F4/80 (macrophage marker) or CD11b marker of leukocytes (including monocytes, neutrophils, natural killer cells, granulocytes and macrophages) positive cell types isolated from the spleen in either the CTL mice treated with tamoxifen or the VEGFR2^ECKO^ mice (Fig. 9).

## Discussion

We show here that the microvasculature in the spinal cord was disrupted in a rodent model of diabetic neuropathic pain, as demonstrated by a reduction in the volume of the blood vessel network in the spinal cord. These findings are associated with neuropathic pain development and spinal neuronal activation. There was a concurrent reduction in spinal cord expression of the VEGF‐A_165_b isoform, but no overall change in total VEGF‐A expression. In the inducible VEGFR2^ECKO^ mice, the vasculature in the lumbar spinal cord was also reduced and was accompanied by the development of hyperalgesia. Critically, reversing the diabetes‐induced vascular degeneration using systemic VEGF‐A_165_b treatment also resulted in reversal of chronic pain.

Neuropathic pain is common in diabetic patients and is typically associated with hyperglycaemia impacting upon the peripheral vasculature through alterations in blood flow and solute leakage (Poduslo & Curran, 1992; Tesfaye *et al*. 2013; Hulse *et al*. 2015). The peripheral sensory nerves are compromised in these instances through a degeneration of nerve terminals (loss of intraepidermal nerve fibre innervation) in the skin (Narayanaswamy *et al*. 2012), atrophy of the nerve trunk (shrinkage of nerve fibre diameter) (Hulse *et al*. 2015), reduction in nerve conduction velocity (Ali *et al*. 2014) and excitation of C nociceptor fibres (Chen & Levine, 2001). However, despite these widely diagnosed symptoms, current treatments have poor success rates and low long‐term success. Recent advances in understanding diabetic pain have highlighted that neuropathic pain is associated with activation and alteration of nociceptive processing in the CNS. The spinal cord (Morgado *et al*. 2010) and brain (Silva *et al*. 2013) are hyperactivated in rodent models of diabetic neuropathic pain, with increased activity of spinal neurons of wide dynamic range (Pertovaara *et al*. 2001; Morgado *et al*. 2010; Tan *et al*. 2012). Microvascular degeneration is disrupted in the brain of diabetic rodents and linked to, for example, cognitive impairment (Taylor *et al*. 2015; Hardigan *et al*. 2016), although the impact of hyperglycaemia on the *spinal cord* microvasculature has not been reported previously. The results we present here support the conclusion that there is a widespread vasculopathy in the spinal cord, which is associated with diabetes and nociceptive processing. The reduced spinal vasculature associated with hyperglycaemia could be in part due to an increase in endothelial cell death. This compromises the function of the spinal cord microcirculation – to provide delivery of solutes as well as appropriate endothelial cell signalling – as evidenced by altered neuronal activation (c‐fos staining) in all five lamina of the dorsal horn, and caspase staining in laminae 1 and 2. Whether the effect on sensory neuronal function within the spinal cord is impaired as a result of decreased nutrient/oxygen delivery or through disturbance of an endothelial–neuronal–glial signalling event remains to be determined.

Multiple diabetic complications arise due to vascular pathology whereby vessel or cell function is impaired (e.g. retinopathy, nephropathy) (Gupta *et al*. 2013; Oltean *et al*. 2014). We have previously shown that enhanced extravasation in diabetes does occur in the peripheral nervous system, including in the DRG, and this is mediated in part through VEGF‐A, as treatment with VEGF‐A_165_b (which can inhibit endogenous VEGF‐A_165_b signalling through VEGFR2, and prevent TRPA1 and TRPV1 activation in cultured DRG cells *ex vivo*) blocked the enhanced extravasation and the associated pain behaviours. In this case we describe a *reduction* in solute flux in the CNS, which combined with a loss of vessels can only be attributed to a reduction in blood flow. Evans blue extravasation is often taken as an indication of increased vascular permeability, and if blood is still flowing to the vessels where the vascular permeability increases, this is often consistent with the data. However, extravasation requires a provision of solute in the blood, and therefore is highly dependent on blood flow. A *reduction* in Evans blue extravasation in the spinal cord, where the permeability is already very low compared with most tissues (e.g. heart, skin, DRG; Hulse *et al*. 2015), can only be reasonably interpreted as an indication of reduced surface area for exchange, or reduced capillary pressure. The loss of endothelial cell staining, reduction of CD31 combined with reduced Evans blue extravasation indicates that diabetic animals have lost functional blood vessels from the spinal cord. This is an interesting comparison with most other vascular beds [including eyes (Ved *et al*. 2017) and the peripheral nerves (Hulse *et al*. 2015)], where diabetes results in increased solute flux, probably through increased vascular permeability (Poduslo & Curran, 1992). In peripheral tissues such as in the sensory nerve and the DRG, increased blood–nerve barrier breakdown could arise due to direct glucose toxicity upon the vessels in the epineurium. However, sensory nerves are activated following exposure to high blood glucose (Chen & Levine, 2001; Chen & Levine, 2003). Such activity would drive peripheral extravasation through release of CGRP or activation of TRPA1; this is activated in sensory nerves of diabetic rodents (Koivisto *et al*. 2012; Hulse *et al*. 2015). These systems are not expected to be in play in the spinal cord so alternative mechanisms must be in action.

In this study we find that alterations in VEGF‐A/VEGFR2 activation, as evidenced both by inhibition of VEGFR2 by VEGF‐A_165_b (Bates *et al*. 2002; Cébe Suarez *et al*. 2006) and by VEGFR2 knockout, affect the decline in microvascular function. The VEGF‐A family consists of two families of alternative spliced isoforms termed VEGF‐A_xxx_a and VEGF‐A_xxx_b (xxx denote amino acid number). These isoforms differ solely due to exon 8 splicing giving rise to differing C terminus sequences that critically alter isoform function (Harper & Bates, 2008). It has been shown that both bind to VEGFR2 with equal affinity and both possess cytoprotective actions, however VEGF‐A_xxx_a is pro‐angiogenic and VEGF‐A_xxx_b is able to inhibit VEGF‐A_xxx_a mediated angiogenesis (Woolard *et al*. 2004). Therefore VEGF‐A_165_b (acting via VEGFR2) may act as a vascular protective agent under normal conditions, preventing loss of blood vessels. Loss of VEGF‐A_165_b in diabetic spinal cord removes such protection, resulting in vascular damage, and reducing both vascular integrity and function. This concept, that loss of endogenous endothelial cell VEGFR2‐mediated cytoprotection contributes to nociceptive processing, is consistent with the results from the endothelial‐specific VEGFR2 KO mice. These mice demonstrated loss of spinal cord endothelium and associated development of hyperalgesia, consistent with the concept that endogenous maintenance of VEGFR2 activity is cytoprotective to the spinal cord endothelium, albeit that we have not directly measured spinal cord neuronal nociceptive processing. One interesting difference between the diabetic and VEGFR2^ECKO^ animals was that whereas the diabetic rats had a reduction in larger microvessels and no change in smaller vessels, the VEGFR2^ECKO^ animals had increased numbers of small but reduced numbers of larger vessels. This suggests that in diabetes the loss of vessels was due to either selective loss of larger microvessels (8–12 μm) or a combination of loss of all vessels and a reduction in size of the larger vessels (hypotrophy or atrophy, or vasoconstriction). In contrast, the VEGFR2^ECKO^ mice showed the latter effect. The functional implications would still be ischaemia, but may have other subtle differences in nutrient or cell delivery. While our understanding of spinal nociceptive neuronal processing in diabetes is progressing, the contribution of microvascular alteration in this context has not been investigated. Furthermore, it has been shown that peripheral sensory neurons become sensitised upon reduced perfusion (So *et al*. 2016), leading us to speculate that the changes in the spinal cord vascular network, as a result of hyperglycaemia that we report, could alter the microenvironment of the spinal cord sensory neurons and thus alter their level of activation. This would be anticipated to contribute to changes in pain perception and underlie neuropathic pain development.

The VEGFR2 knockdown was restricted to Tie2‐positive cell types, which encompass the endothelial and haemopoietic cell populations. The Tie2 promoter‐driven transgenic models are widely used to investigate endothelial function (Makino *et al*. 2014; Moyes *et al*. 2014), although it has been reported that Tie2 is expressed on CD11b/CD45‐positive cells. (De Palma *et al*. 2005; Tang *et al*. 2010). In this study there was no impact of VEGFR2 knockout on haematopoietic cell populations. An impact of this knockout system on circulatory macrophages and consequent endothelium would be expected to be minimal as there is a very small number of, if any, Tie2‐positive, VEGFR2‐positive macrophages (Okubo *et al*. 2016).

People with diabetes are susceptible to neurological disease as a result of microvascular dysfunction, impacting on motor, autonomic and cognitive systems, and increased susceptibility to stroke and dementia (Kissela *et al*. 2005; Tiehuis *et al*. 2008). The VEGF‐A angiogenic family has a pivotal role in managing vascular development and function (Bates, 2010), but additional evidence now also supports a fundamental role for this family in neuronal activity and survival (Verheyen *et al*. 2012; Beazley‐Long *et al*. 2013). As a consequence, VEGF‐A treatment has been trialled for peripheral diabetic neuropathy (Schratzberger *et al*. 2001; Ropper *et al*. 2009). Experimental hyperglycaemia leads to a reduction in VEGF‐A and VEGFR2 expression in the brain; VEGF‐A treatment can protect both endothelium and neuronal circuits (Taylor *et al*. 2015). Dysfunction of the VEGF‐A signalling pathway within the hippocampus impacts upon the microvasculature and leads to impaired spatial memory (Reeson *et al*. 2015; Taylor *et al*. 2015). A decline in vascular support and diminished vascular response in diabetes and stroke models therefore clearly affects neuronal function. However, VEGF‐A_165_a also increases vascular permeability and stimulates abnormal angiogenesis, which is detrimental in the CNS, for instance in diabetic retinopathy (Perrin *et al*. 2005). The VEGF‐A_xxx_b isoforms, however, do not stimulate angiogenesis or increase solute flux in diabetes, and in fact they can reverse it in rodent models of diabetic retinopathy (Ved *et al*. 2017). Despite these opposing profiles both families are cytoprotective for endothelial cells and neurons (Beazley‐Long *et al*. 2013; Oltean *et al*. 2014; Hulse *et al*. 2015; Vencappa *et al*. 2015). For example, reduced VEGF‐A_165_b expression in diabetic patients is associated with reduced kidney function (Oltean *et al*. 2014). Furthermore, treatment of diabetic mice with VEGF‐A_165_b prevented endothelial dysfunction in the kidney of these animals (Oltean *et al*. 2014). Here we show that a reduction in VEGF‐A_165_b in the lumbar spinal cord of diabetic animals was associated with neuropathic pain and a degeneration of the spinal vasculature. Systemically reintroducing VEGF‐A_165_b prevented vascular degeneration, spinal cord neuron activation and pain. VEGF‐A_165_b was reduced in diabetic rats, despite reports that VEGF‐A_165_b is associated with inhibition of vessel growth (Woolard *et al*. 2004). Thus VEGF‐A_165_b should be considered both an anti‐angiogenic and an endothelial survival factor – or a homeostatic counterpart to its vascular remodelling isoform, VEGF‐A_165_a. It must be noted that the actions of VEGF‐A_165_b and VEGFR2 on pain may not be restricted to vascular protection but also to effects on the spinal cord neurons, either indirectly, as VEGF‐A_165_b has been shown to inhibit peripheral sensory neuron excitability and activation of peripheral nociceptors induces c‐fos expression in the dorsal horn (Hulse *et al*. 2014), or directly through inhibiting VEGF‐A_165_a actions on spinal cord circuitry (Hulse *et al*. 2016). Accompanying this, neutralising endogenous VEGF‐A_165_b as well as pharmacological blockade of VEGFR2 signalling at the level of the spinal cord led to the development of pain (Hulse *et al*. 2016). In addition, reductions in VEGF‐A_165_b expression and no change in total VEGF‐A expression would highlight a plausible increase in alternative isoforms such as VEGF‐A_165_a, an event associated with pro‐nociception and chronic pain (Hulse *et al*. 2014, 2016), which cannot be ruled out in this instance.

Furthermore, some consideration does need to be made when using diabetic models such as STZ. STZ is a reliable rodent model of type 1 diabetic neuropathy displaying comparable pain behaviour, nerve electrophysiological parameters and nerve histology, used widely throughout the field of diabetes research. However, it is an experimental model of islet cell ablation by a toxic agent that may have other effects, so despite its widespread use in many experimental models of diabetic complications (nephropathy, neuropathy, retinopathy and others) it is still critical that these findings are reproduced in type II diabetic models and other models of type I diabetes before being applied to people with diabetes.

These findings lead us to speculate that diabetes‐induced alterations of the somatosensory systems, for instance by affecting the vasculature supporting somatosensory processing at the level of the spinal cord, could be a key concept in regulating neuropathic pain, and treatment of such complications (e.g. spinal cord vasculopathy) could provide a key target in treating diabetic neuropathic pain.

In summary, we report for the first time a significant vascular degeneration in the spinal cord of diabetic rats, which is associated with a loss of spinal VEGF‐A_165_b. This was accompanied by spinal neuron activation, indicative of altered function of the spinal cord neurons, and also by enhanced nociceptive pain behaviour. Administration of VEGF‐A_165_b alleviated both the spinal cord vascular degeneration and neuropathic pain. This work is complementary to previous work in the nervous system demonstrating a neuroprotective action of VEGF‐A (Reeson *et al*. 2015; Taylor *et al*. 2015), but here we demonstrate central effects in the spinal cord with potential contribution to the control of chronic pain development, rather than actions purely at peripheral sites. These findings provide additional avenues for further understanding diabetic neuropathic pain and the possible mechanisms that underlie sensitisation of nociceptive pathways.

## Additional information

### Competing interests

LFD and DOB are co‐inventors on patents protecting VEGF‐A_165_b and alternative RNA splicing control for therapeutic application in a number of different conditions. LFD and DOB are founder equity holders in, and DOB is a director of Exonate Ltd, a company with a focus on the development of alternative RNA splicing control for therapeutic application in a number of different conditions, including diabetic complications. The authors have no other conflicts of interest to declare.

### Author contributions

NV, RPH, MD, NBL, CV and SMB performed the animal work, the immunofluorescence, cell culture and immunoblotting in the Tumour and Vascular Biology Laboratories at the University of Nottingham. KBH generated the recombinant human VEGF‐A_165_b protein. RPH, NV, NBL, CV, MH, KBH, MD, SMB, LFD and DOB contributed to the conception or design of the work in addition to acquisition, analysis or interpretation of data for the work. RPH, NV, NBL, CV, MH, KBH, MD, SMB, LFD and DOB drafted the work or revised it critically for important intellectual content. RPH drafted the manuscript with contributions from all authors. All authors approved the final version of the manuscript, agree to be accountable for all aspects of the work in ensuring that questions related to the accuracy or integrity of any part of the work are appropriately investigated and resolved. All persons designated as authors qualify for authorship, and all those who qualify for authorship are listed.

### Funding

This work was supported by the Medical Research Council [grant number MR/K013157/1]; Arthritis Research UK [grant number 20400]; Diabetes UK [11/0004192, 10/0004152]; The Richard Bright VEGF Research Trust (UK Registered Charity 1095785); the European Foundation for the Study of Diabetes Microvascular Programme supported by Novartis and Rosetrees Trust.
